# Diagnostic Utility of Quantitative Strain Elastography in Breast Lesions: A Comparative Evaluation of Distance Ratio and Strain Ratio

**DOI:** 10.7759/cureus.118871

**Published:** 2026-10-31

**Authors:** Preeti Yadav, Arindam Mukherjee, Riya Samanta

**Affiliations:** 1 Radiology, World College of Medical Sciences and Research, Jhajjar, IND; 2 Radiology, BLK-Max Super Speciality Hospital, New Delhi, IND; 3 Radiodiagnosis, Lady Hardinge Medical College, New Delhi, IND

**Keywords:** breast cancer, breast lesions, breast ultrasonography, distance ratio, histopathology, strain elastography, strain ratio

## Abstract

Introduction

Accurate characterization of breast masses is essential for appropriate clinical management and reduction of unnecessary invasive procedures. Quantitative strain elastography is a useful adjunct to conventional breast ultrasonography by providing an objective assessment of tissue stiffness. Although the strain ratio (SR) has been widely investigated, the diagnostic utility of the distance ratio (DR) remains relatively underexplored. This study aimed to evaluate the diagnostic utility of quantitative strain elastography in differentiating benign from malignant breast lesions, with particular emphasis on the comparative performance of the DR and SR, using histopathology as the reference standard.

Methods

This prospective cross-sectional study included 30 women presenting with palpable breast masses or lesions detected on sonomammography. All patients underwent conventional breast ultrasonography followed by quantitative strain elastography. The DR and SR were recorded and compared with histopathological findings. Receiver operating characteristic (ROC) analysis was performed to determine optimal cutoff values and evaluate the diagnostic performance of both parameters.

Results

Histopathological examination revealed 20 (66.7%) malignant and 10 (33.3%) benign breast lesions. The mean DR was significantly higher in malignant lesions than in benign lesions (1.60 ± 0.27 vs. 1.06 ± 0.11; p < 0.001). At a cutoff value of 1.23, the DR demonstrated 95% sensitivity, 100% specificity, 100% positive predictive value (PPV), 90.9% negative predictive value (NPV), and an overall diagnostic accuracy of 96.7%. The mean SR was also significantly higher in malignant than in benign lesions (7.41 ± 3.73 vs. 2.21 ± 1.05; p < 0.001). At a cutoff value of 3.61, the SR demonstrated 95% sensitivity, 90% specificity, and an overall diagnostic accuracy of 93.3%.

Conclusions

Both DR and SR demonstrated promising diagnostic performance in differentiating benign from malignant breast lesions in this cohort. Quantitative strain elastography may serve as an adjunct to conventional breast ultrasonography; however, the findings do not establish the superiority of either parameter. Larger studies involving independent cohorts are needed to validate the derived cutoff values and determine the reproducibility and clinical applicability of these parameters.

## Introduction

Breast cancer is the most commonly diagnosed cancer among women worldwide as well as an important cause of cancer-related mortality in both developing and developed countries. Appropriate and early characterization of breast masses is important for clinical decision-making, which may help guide decisions regarding biopsy. Mammography is the primary imaging modality for breast cancer screening, whereas ultrasonography is widely used for the evaluation of palpable breast abnormalities, particularly in women with dense breasts [1-3].

B-mode ultrasonography provides information about lesion morphology, vascularity, and associated findings. Benign and malignant lesions may show overlapping morphological features, making further imaging evaluation or biopsy necessary in indeterminate cases [4].

Strain elastography provides information about tissue stiffness by evaluating tissue deformation in response to externally applied compression. Malignant breast lesions are generally stiffer than benign lesions due to differences in cellularity, fibrosis, and tissue composition. Studies suggest that elastography combined with conventional ultrasonography may improve breast lesion characterization, specificity, and diagnostic confidence [5-9].

The strain ratio (SR) is a widely studied quantitative parameter that compares lesion stiffness with reference tissue. Higher values are generally associated with malignancy, although reported cutoffs vary across studies due to differences in technique, equipment, and lesion characteristics [10,11].

The distance ratio (DR) or elastogram-to-B-mode ratio compares the linear lesion dimension on elastography or length with the corresponding dimension on B-mode ultrasonography. Higher ratios are generally associated with malignancy and reflect stiffness extending beyond the visible lesion. Unlike the SR, it does not require a separate reference tissue, making it a more practical parameter for lesion assessment [12].

Although SR has been extensively studied, fewer studies have evaluated DR for differentiating benign from malignant breast masses. Furthermore, evidence directly comparing the diagnostic performance of both parameters in the same patient population remains limited. Since DR and SR differ in their measurement requirements and may provide complementary diagnostic information, their comparative evaluation may help clarify their respective diagnostic utility.

Therefore, this study aimed to evaluate and compare the diagnostic performance of DR and SR derived from quantitative strain elastography in differentiating benign from malignant breast masses, using histopathological diagnosis as the reference standard.

## Materials and methods

Study design, patient population, inclusion and exclusion criteria

This prospective observational study involving 30 female patients with breast masses was conducted at World College of Medical Sciences & Research and Hospital, Jhajjar, Haryana, India, between April 2026 and July 2026, following approval from the Institutional Ethics Committee. Eligible patients were consecutively recruited during the study period according to the predefined inclusion and exclusion criteria. No formal a priori sample-size calculation was performed.

The inclusion criteria were female patients presenting with a palpable breast lump and female patients with a breast lesion detected on imaging. The exclusion criteria were patients with a history of breast surgery involving the same breast and patients with a history of chemotherapy or radiotherapy for breast lesions.

Imaging protocol

After obtaining written informed consent, all patients underwent B-mode ultrasonography followed by strain elastography. Imaging findings were correlated with the final pathological diagnosis, which served as the reference standard. The radiologist performing the ultrasound and elastography examinations was blinded to the histopathological findings at the time of image acquisition and measurement.

Ultrasound examination

All examinations were performed using a Siemens ACUSON S2000 ultrasound system (Siemens Healthineers, Erlangen, Germany) equipped with a 9L4 linear-array transducer (4-9 MHz). All examinations were performed by a radiologist with seven years of experience in diagnostic radiology.

Patients were examined in the supine or supine-oblique position with the ipsilateral arm elevated above the head. Adequate coupling gel was used, the transducer was kept perpendicular to the skin surface, and a stable grayscale image was obtained.

Lesions were assessed for size, shape, orientation, margins, echogenicity, posterior acoustic features, calcification, nipple retraction, ductal dilatation, and skin thickening.

Quantitative strain elastography

Quantitative strain elastography was performed using the same ultrasound system, with the same depth, focus, and gain settings and patient positioning throughout the study. Elastographic images were acquired using the freehand manual compression technique, with gentle, repetitive vertical compression applied while maintaining the transducer perpendicular to the lesion. A consistent acquisition technique was maintained throughout the examinations. A split-screen display was used to simultaneously visualize the lesion on B-mode ultrasonography and the corresponding elastographic image.

A single measurement of each quantitative elastography parameter was recorded for each lesion and used for statistical analysis. Two quantitative parameters were assessed: DR and SR.

Distance Ratio (DR)

The DR, also described as the elastogram-to-B-mode ratio or length ratio, was calculated by comparing the linear dimension of the lesion measured on the strain elastogram with the corresponding linear dimension measured on the B-mode image in the same imaging orientation [13]. The mirror function was used, in which the lesion was measured on the B-mode image and the size measurement was automatically depicted on the elastographic image. The DR was calculated using the following formula: $\mathrm{DR} = \frac{\text{Elastographic lesion dimension}}{\text{B-mode lesion dimension}}$.

Strain Ratio (SR)

For SR measurement, two manually selected regions of interest (ROIs) were placed on the elastograms. One ROI was placed within the solid component of the lesion, and the second was placed in adjacent normal-appearing breast parenchyma at a similar depth [11]. Areas of cystic degeneration, necrosis, and calcification were excluded while placing the ROIs.

The SR was calculated automatically by the elastographic software as the ratio of the strain measured in the surrounding reference tissue to the strain measured within the lesion.

Pathological evaluation

The final diagnosis of all 30 breast lesions was established by histopathological examination of specimens obtained through core needle biopsy or surgical excision, as clinically indicated. Histopathological findings served as the reference standard for classifying each lesion as benign or malignant.

Statistical analysis

Categorical variables were expressed as frequencies and percentages, while continuous variables were summarized as mean ± standard deviation and median, as appropriate. The DR and SR were compared between benign and malignant lesions, and the corresponding p-values were calculated. Receiver operating characteristic (ROC) curve analysis was performed using the study data to evaluate the diagnostic performance of DR and SR and to derive cutoff values for differentiating benign from malignant breast lesions. Diagnostic performance was assessed using sensitivity, specificity, positive predictive value (PPV), negative predictive value (NPV), and diagnostic accuracy. A two-sided p-value of less than 0.05 was considered statistically significant. Statistical analyses were performed using IBM SPSS Statistics for Windows, Version 21.0 (Released 2012; IBM Corp., Armonk, NY, USA).

## Results

Patient characteristics

Thirty female patients with breast lesions were included in the study, with ages ranging from 18 to 64 years. The demographic characteristics are summarized in Table 1.

**Table 1:** Baseline Demographic Characteristics

Characteristic	Value
Total patients	30
Mean age (years)	40.66 (18-64)
Benign lesions, n (%)	10 (33.3)
Malignant lesions, n (%)	20 (66.7)
Mean age of benign group (years)	24.7
Mean age of malignant group (years)	48.7

Pathological findings

Final pathological evaluation diagnosed 20 (66.7%) malignant and 10 (33.3%) benign lesions. Invasive ductal carcinoma was the most common malignant diagnosis, and fibroadenoma was the most frequent benign diagnosis. The final pathological diagnoses are presented in Table 2.

**Table 2:** Final Pathological Diagnosis

Diagnosis	Number (%)
Malignant lesions (n = 20)	
Invasive ductal carcinoma	16 (80)
Ductal carcinoma in situ	1 (5)
Metaplastic carcinoma	1 (5)
Intraductal papillary carcinoma	1 (5)
Invasive lobular carcinoma	1 (5)
Benign lesions (n = 10)	
Fibroadenoma	8 (80)
Pseudoangiomatous stromal hyperplasia (PASH)	1 (10)
Breast abscess	1 (10)

Quantitative strain elastography

Both quantitative elastographic parameters were significantly higher in malignant lesions than in benign lesions (p < 0.001 for both).

The DR ranged from 1.07 to 2.16 in malignant lesions and from 0.88 to 1.19 in benign lesions (Figures 1-2). The mean DR was significantly higher in malignant lesions than in benign lesions (1.60 ± 0.27 vs. 1.06 ± 0.11; p < 0.001). ROC analysis yielded an area under the curve (AUC) of 0.970. At a cutoff value of 1.23, DR demonstrated 95% sensitivity, 100% specificity, and 96.7% diagnostic accuracy (Tables 3-4).

**Figure 1:**
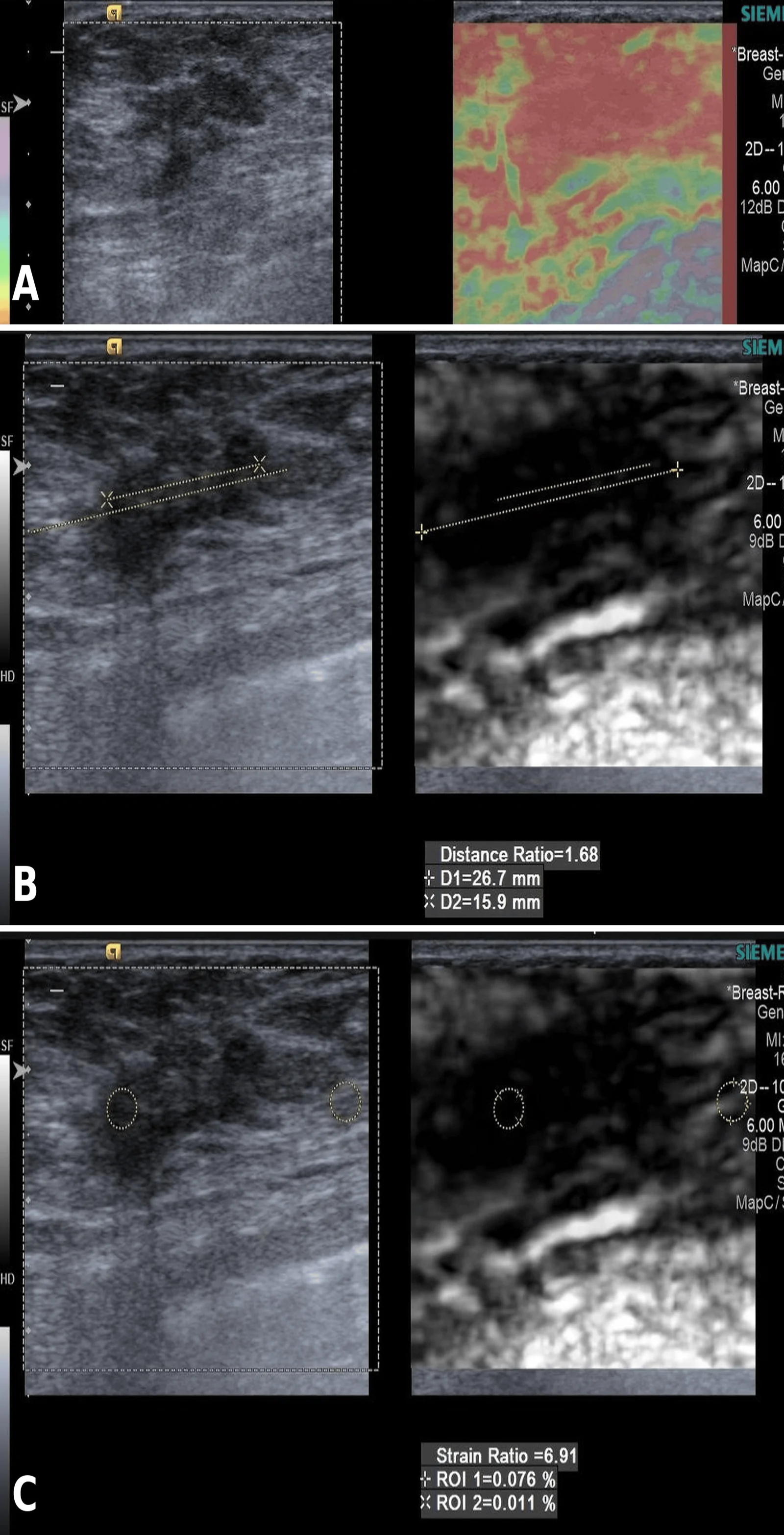
Strain Elastography of Histopathologically Confirmed Invasive Ductal Carcinoma of the Breast (A) B-mode USG image shows an irregular hypoechoic lesion with spiculated margins, and the corresponding color elastogram shows increased stiffness, depicted by red color throughout the mass. (B) Distance ratio measurement on strain elastography, obtained by comparing the lesion dimension on the elastographic image (D1 = 26.7 mm) with that on the corresponding B-mode image (D2 = 15.9 mm), showed a value of 1.68. (C) Strain ratio measurement on strain elastography, calculated as the ratio of strain generated in the region of interest (ROI) within the lesion (ROI1 = 0.076%) to that in the second ROI in the surrounding reference breast tissue (ROI2 = 0.011%), showed a value of 6.91.

**Figure 2:**
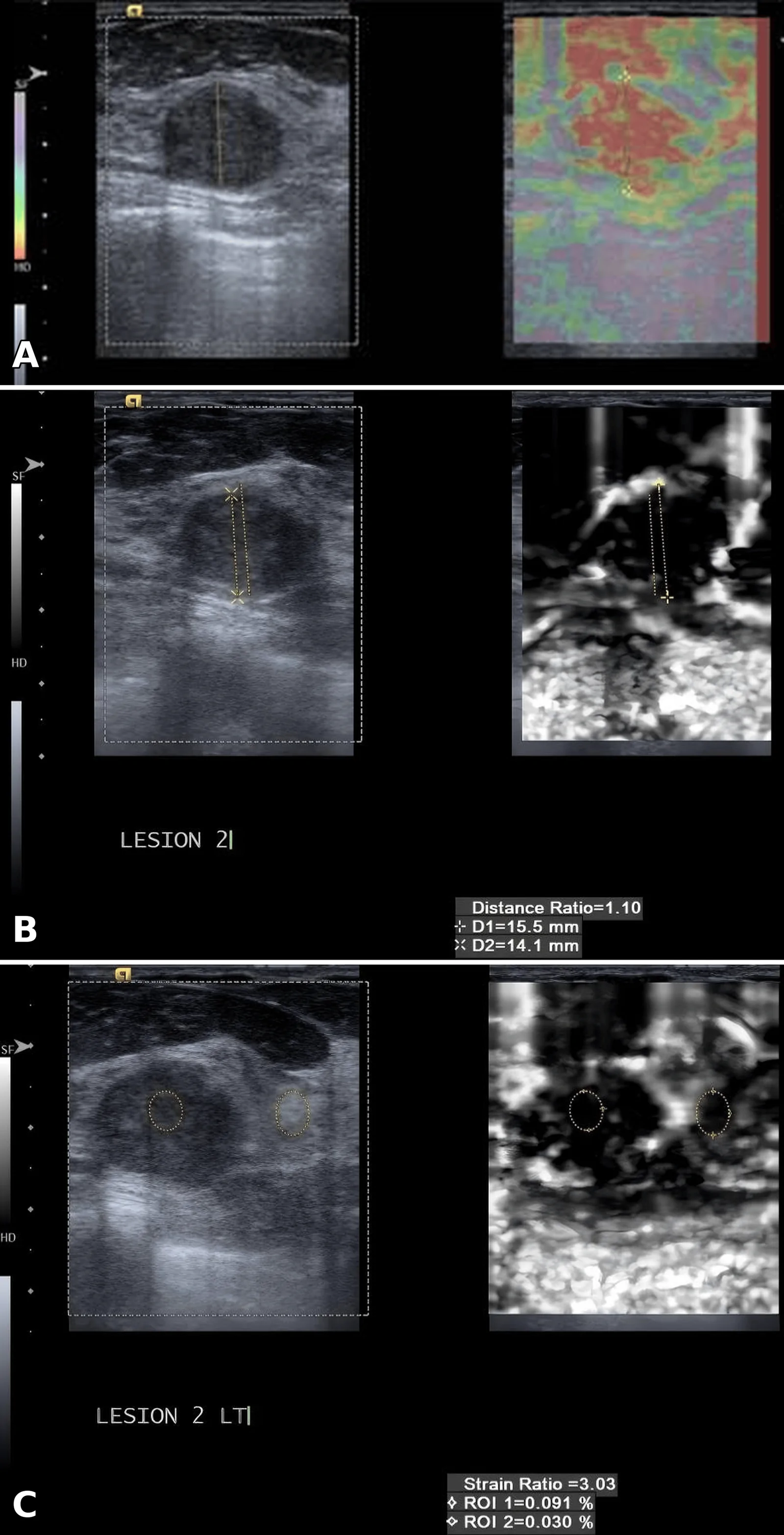
Strain Elastography of Histopathologically Confirmed Fibroadenoma (A) B-mode USG image shows an oval, wider-than-tall hypoechoic lesion with circumscribed margins, and the corresponding color elastogram shows a heterogeneous pattern, depicted by areas of red intermixed with green and blue within the mass. (B) Distance ratio measurement on strain elastography, obtained by comparing the lesion dimension on the elastographic image (D1 = 15.5 mm) with that on the corresponding B-mode image (D2 = 14.1 mm), showed a value of 1.10. (C) Strain ratio measurement on strain elastography, calculated as the ratio of strain generated in the region of interest (ROI) within the lesion (ROI1 = 0.091%) to that in the second ROI in the surrounding reference breast tissue (ROI2 = 0.030%), showed a value of 3.03.

**Table 3:** Comparison of Quantitative Strain Elastography Parameters According to Pathological Diagnosis

Parameter	Benign (n=10), Mean ± SD	Benign range	Malignant (n=20), Mean ± SD	Malignant range	p-value
Distance ratio	1.06 ± 0.11	0.88-1.19	1.60 ± 0.27	1.07-2.16	<0.001
Strain ratio	2.21 ± 1.05	0.71-3.98	7.41 ± 3.73	3.38-14.08	<0.001

**Table 4:** Diagnostic Performance of Quantitative Strain Elastography

Parameter	Distance ratio	Strain ratio
Cutoff value	1.23	3.61
Area under the receiver operating characteristic curve (AUC)	0.970	0.980
Sensitivity (%)	95	95
Specificity (%)	100	90
Positive predictive value (%)	100	95
Negative predictive value (%)	90.9	90
Diagnostic accuracy (%)	96.7	93.3

The SR ranged from 3.38 to 14.08 in malignant lesions and from 0.71 to 3.98 in benign lesions (Figures 1-2). The mean SR was higher in malignant lesions (7.41 ± 3.73) than in benign lesions (2.21 ± 1.05), and the difference was statistically significant (p < 0.001). ROC analysis showed an AUC of 0.980. Using a cutoff of 3.61, SR achieved a sensitivity of 95%, specificity of 90%, and overall diagnostic accuracy of 93.3% (Tables 3-4).

Comparative diagnostic performance

Both DR and SR demonstrated excellent diagnostic discrimination between benign and malignant breast lesions in this study. SR had a slightly higher AUC than DR (0.980 vs. 0.970), whereas DR demonstrated higher specificity (100% vs. 90%) and overall diagnostic accuracy (96.7% vs. 93.3%).

False-positive and false-negative findings

At the selected cutoff values, both DR and SR yielded one false-negative result, in which a malignant lesion was classified as benign. DR yielded no false-positive results, whereas SR yielded one false-positive result, in which a benign lesion was classified as malignant. These findings indicate that both parameters may occasionally misclassify breast lesions despite their high overall diagnostic performance.

## Discussion

Our study evaluated two quantitative parameters of strain elastography, namely the DR and SR, in 30 breast lesions. Both parameters were significantly higher in malignant lesions than in benign lesions, supporting the potential role of quantitative strain elastography as an adjunct to conventional B-mode ultrasonography. Previous studies have similarly reported that elastography provides complementary information about tissue mechanical properties and may improve breast lesion characterization [5-9].

An important strength of this study was the assessment of both DR and SR in the same cohort of patients, allowing their diagnostic performance to be evaluated within the same study population.

The desmoplastic stromal reaction associated with malignant lesions may cause the stiff region on elastography to extend beyond the margins identified on B-mode imaging, resulting in higher DR. In our study, the mean DR was 1.60 ± 0.27 in malignant lesions and 1.06 ± 0.11 in benign lesions. ROC analysis yielded an AUC of 0.970, with a cutoff value of 1.23 providing a sensitivity of 95% and specificity of 100%. The corresponding PPV, NPV, and diagnostic accuracy were 100%, 90.9%, and 96.7%, respectively. However, as both DR and SR were evaluated individually without a formal statistical comparison between them, the relative diagnostic performance of the two parameters remains undetermined.

The two parameters evaluated in this study assess different aspects of lesion characteristics. The SR reflects relative tissue stiffness, whereas the DR reflects the difference in apparent lesion extent between elastographic and B-mode imaging. The DR is calculated from lesion dimensions on elastographic and B-mode images without requiring a separate reference ROI, which may offer a relatively straightforward approach to quantitative assessment in clinical practice.

Furthermore, the relatively small sample size and the derivation and evaluation of the ROC-based cutoff values within the same cohort may have resulted in optimistic estimates of diagnostic performance. Although both parameters demonstrated promising results, the observed AUC values do not establish the superiority of either parameter.

One case of invasive lobular carcinoma showed a false-negative DR of 1.07, below the cutoff of 1.23. This may be related to the infiltrative growth pattern of invasive lobular carcinoma, in which tumor cells infiltrate the breast tissue without necessarily forming a well-defined mass. Consequently, the difference between lesion dimensions on elastography and B-mode imaging may be less pronounced, resulting in a lower DR. Further studies may help clarify this association.

The use of lesion size differences between elastographic and B-mode images has been described in previous studies. Sadigh et al. evaluated breast lesions using length ratio and reported a sensitivity of 98%, which was broadly comparable to the sensitivity observed in our study [14]. Barr and Zhang reported a mean elastography-to-B-mode (E/B) ratio of 1.67 ± 0.46 in malignant breast lesions, comparable to the mean DR of 1.60 ± 0.27 in our study [15]. 

The SR was the second quantitative parameter evaluated in our study. Malignant lesions generally have higher SR values because of increased stiffness associated with desmoplastic reaction and tumor-induced fibrosis, resulting in reduced deformation under compression compared with the reference tissue.

The SR also demonstrated high diagnostic performance, with a mean value of 7.41 ± 3.73 in malignant lesions compared with 2.21 ± 1.05 in benign lesions (p < 0.001). The AUC was 0.980, and a cutoff of 3.61 yielded a sensitivity of 95%, specificity of 90%, PPV of 95%, NPV of 90%, and diagnostic accuracy of 93.3%. These results should be interpreted cautiously, as the cutoff was derived and evaluated in the same cohort.

One fibroadenoma showed a false-positive SR of 3.98, which may be related to stromal fibrosis or hyalinization. In contrast, the invasive lobular carcinoma had a false-negative SR of 3.41. This may be due to a less marked desmoplastic response, resulting in a smaller difference in stiffness between the lesion and the reference tissue. This lesion, missed by both DR and SR, was also indeterminate on B-mode ultrasound. Thus, elastography did not resolve the diagnostic uncertainty in this case, highlighting that quantitative elastography may not overcome the limitations of conventional ultrasound in every indeterminate lesion and should be interpreted in conjunction with morphological findings and clinical assessment.

The diagnostic performance of strain elastography in differentiating benign from malignant breast lesions has been evaluated in several studies. Khamis et al. [16], in a study involving 120 breast masses, reported a mean SR of 6.91 ± 3.96 for malignant lesions and 2.12 ± 1.72 for benign lesions. Similarly, Zhao et al. [17], in a study of 187 solid breast lesions in 155 patients, found that the mean SR was significantly higher in malignant lesions (6.66 ± 4.62) than in benign lesions (2.06 ± 1.27).

The SR cutoff of 3.61 identified in our study is comparable to the cutoff of 3.50 reported by Bojanic et al. [18] for differentiating benign from malignant breast lesions. Mutala et al., in a systematic review and meta-analysis of 65 studies, found substantial variation in reported SR cutoffs related to differences in equipment, reference-tissue selection, lesion characteristics, and study populations [19].

Quantitative strain elastography may provide complementary information about tissue stiffness when used alongside conventional breast imaging. However, the present study did not evaluate its effect on biopsy recommendations or clinical outcomes; therefore, a reduction in unnecessary biopsies cannot be inferred from these findings.

Further studies are needed to assess its incremental value in clinical decision-making and validate the diagnostic performance of both parameters in larger, independent cohorts.

Limitations

This study had several limitations. The relatively small sample size, including only 10 benign lesions, may limit the precision and generalizability of the findings. The higher proportion of malignant lesions and differences in age distribution between the groups may also have influenced the diagnostic performance estimates. Being a single-center study, its findings may not be generalizable to all clinical settings. Furthermore, the ROC-derived cutoffs were determined and evaluated in the same cohort and require independent validation. Strain elastography is operator-dependent, and interobserver and intraobserver variability were not assessed. Finally, no formal statistical comparison between DR and SR was performed; therefore, the study does not establish the superiority of either parameter.

## Conclusions

Quantitative strain elastography may complement conventional B-mode ultrasonography in the characterization of breast lesions. Both DR and SR demonstrated promising diagnostic performance in this cohort, although the findings do not establish the superiority of either parameter. Further studies are needed to determine their reproducibility and clinical applicability.
